# Handcrafted Versus Deep Feature Extraction Methods for MRI-Based Multiple Sclerosis Diagnosis

**DOI:** 10.3390/diagnostics16091379

**Published:** 2026-05-01

**Authors:** Samah Yahia, Tahani Bouchrika, Wided Bouchelligua

**Affiliations:** 1 Research Laboratory Modeling, Analysis and Control of Systems (MACS), National Engineering School of Gabes (ENIG), Gabes 6029, Tunisia; ysamah4@gmail.com; 2 Computer Science Department, University College of Haql, University of Tabuk, Tabuk 71491, Saudi Arabia; tboushrika@ut.edu.sa; 3 Applied College, Imam Mohammad Ibn Saud Islamic University (IMSIU), Riyadh 11432, Saudi Arabia

**Keywords:** MS detection, MS progression study, 3D MRI-based diagnosis, DDP-based gradient-enhanced feature extraction, VLM

## Abstract

**Background**: Despite significant advances in medical image analysis, automated diagnosis of Multiple Sclerosis (MS) from magnetic resonance imaging (MRI) remains challenging due to the complexity of 3D brain data and the variability of lesion appearance. **Objective**: In this work, we propose an efficient and optimized feature extraction framework for automated MS diagnosis using FLAIR, T1-, and T2-weighted MRI. The approach enhances Decimal Descriptor Patterns (DDP) by integrating local gradient information, producing a 3D texture representation that is more discriminative and expressive. **Methods**: The study is divided into two main parts: (i) detection of MS, and (ii) assessment of disease progression in affected patients. In each part, features are extracted from the relevant MRI data and classified using multiple classical machine learning classifiers, including Linear Discriminant Analysis (LDA), Support Vector Machines (SVM), k-Nearest Neighbors (KNN), and Logistic Regression. Furthermore, the performance of the proposed handcrafted feature-based approach was compared to features extracted using a deep learning-based model (vision–language model, VLM), specifically CLIP (Contrastive Language–Image Pretraining), enabling a clear comparison of their performance. To assess robustness and generalizability, two complementary validation strategies were adopted: (i) controlled experiments on the BrainWeb dataset under varying T1/T2 contrast conditions, and (ii) validation on a the real-world FLAIR MRI dataset, reflecting clinically relevant lesion visibility. **Results**: Gradient-DDP features achieve the best overall performance for MS progression, reaching up to 97% accuracy on T2-weighted MRI with SVM, while LDA and Logistic Regression also remain strong with accuracies around 83–96% on T2. For binary MS detection, the proposed method attains near-perfect results, with up to 99% accuracy on FLAIR (SVM/KNN) and 98% on T2-weighted images across SVM, while other classifiers also maintain high performance above 90%. **Conclusions**: Gradient-DDP provides strong consistency and transparency, offering an interpretable link between texture patterns and diagnostic outcomes. While VLM features perform well when lesion patterns are clearly defined (e.g., in T2), Gradient-DDP demonstrates greater robustness in more challenging modalities such as Flair, where deep representations may be less stable.

## 1. Introduction

Multiple sclerosis (MS) is a chronic autoimmune disorder of the central nervous system (CNS) characterized by inflammatory demyelination and axonal degeneration, leading to a wide spectrum of neurological symptoms and progressive disability [1,2,3]. The disease arises when the immune system erroneously attacks myelin, the protective sheath surrounding nerve fibers, thereby disrupting neural signal transmission and resulting in focal lesions within the brain and spinal cord [2,3]. Due to the variability in lesion size, location, morphology, and temporal evolution, MS is considered a highly heterogeneous disease, which complicates both diagnosis and long-term monitoring [4,5].

Magnetic resonance imaging (MRI) plays a central role in MS diagnosis and follow-up, as it provides non-invasive visualization of lesion burden and disease activity. In particular, FLAIR, T1-weighted, and T2-weighted MRI sequences offer complementary information regarding lesion visibility, tissue integrity, and structural abnormalities [6,7]. However, detecting MS lesions from 3D MRI volumes remains a challenging computational task due to the high dimensionality of volumetric data, heterogeneous lesion appearances, overlapping brain tissues, and acquisition-related artifacts such as noise and intensity inhomogeneity [8,9,10].

Traditional MS assessment largely relies on visual inspection of MRI scans by expert radiologists, a process that is time-consuming and subject to inter- and intra-observer variability. Subtle or early-stage lesion changes may be overlooked, potentially delaying therapeutic intervention and affecting clinical decision-making. These limitations highlight the need for more sophisticated volumetric feature extraction strategies capable of encoding both local and global lesion properties.

Automated computational approaches have therefore emerged as powerful tools to support MS diagnosis and progression assessment. Feature extraction methods play a pivotal role in this context by transforming complex MRI data into discriminative quantitative descriptors that emphasize lesion boundaries, texture patterns, and intensity variations [10,11,12]. Automated feature extraction methods for MRI-based Multiple Sclerosis (MS) analysis can generally be categorized into two main families: handcrafted feature-based approaches and deep learning-based methods.

Handcrafted approaches rely on the explicit design of descriptors that capture relevant image characteristics such as intensity distributions, texture patterns, shape information, and local structural variations. When combined with classical machine learning classifiers, these features have demonstrated strong capability in accurately differentiating MS from non-MS cases, as well as in monitoring disease progression across time and imaging modalities. Gulay Macin et al. developed an ExMPLPQ handcrafted feature model using local phase quantization with k-nearest neighbor classification, achieving 98.37% accuracy on axial images and 98.22% on hybrid datasets [13]. A. Rezaee et al. employed fractal and Pseudo-Zernike Moment descriptors with an optimized Extreme Learning Machine, obtaining 97% average accuracy in distinguishing MS plaques from comparable lesions [14]. For disease progression monitoring, S. Yahia et al. applied Decimal Descriptor Pattern and Local Binary Pattern operators to characterize brain structures in 3D MRI, demonstrating effective MS evolution tracking [12].

R. Thabet et al. provide a comprehensive review of machine learning and deep learning techniques for multiple sclerosis (MS) classification and segmentation, highlighting that traditional ML methods rely on manual feature extraction, which forms the basis of handcrafted descriptor approaches [15]. In parallel, T. Suryawanshi et al. discuss AI-driven strategies for MS detection and monitoring, emphasizing that machine learning models enhance diagnostic accuracy and facilitate reliable tracking of disease progression, offering robustness, interpretability, and stability essential for clinical applications [16].

In contrast, deep learning-based methods automatically learn hierarchical feature representations directly from data, typically using convolutional neural networks or more recent architectures such as vision–language models (VLMs). These approaches have achieved remarkable performance in various medical image analysis tasks [17,18,19,20,21,22,23]. In this context, several novel VLM architectures have been proposed to better model volumetric medical data. For instance, MS-VLM mimics the radiologist’s workflow by processing 3D medical images slice-by-slice [17]. It employs self-supervised 2D transformer encoders to construct volumetric representations capable of capturing inter-slice dependencies. Building upon this idea, Med3DVLM (2025) introduces three major innovations: a DCFormer encoder based on decomposed 3D convolutions, SigLIP-based contrastive learning, and a dual-stream MLP-Mixer projector. This architecture substantially improves cross-modal retrieval performance, achieving 61.00% R@1 for image–text retrieval compared to 19.10% in previous state-of-the-art models [18].

Similarly, the HiCA framework proposes an adaptive vision–language fine-tuning strategy with hierarchical contrastive alignment to address few-shot medical image classification. This method demonstrates state-of-the-art performance in both few-shot and zero-shot learning scenarios [19]. Collectively, these studies illustrate a clear shift toward multimodal learning strategies that integrate textual and visual medical knowledge.

Parallel to VLM advancements, automated analysis platforms have also evolved rapidly. The Intelligent Healthcare Imaging Platform [20] integrates Google Gemini 2.5 Flash to perform automated tumor detection across multiple imaging modalities, including CT, MRI, X-ray, and ultrasound. Notably, its lesion localization system achieves an average deviation of approximately 80 pixels.

Moreover, CNN-based biomedical analysis systems continue to demonstrate strong performance, with evidence that convolutional neural networks can automatically extract discriminative features from medical images, providing faster and more consistent outputs than manual procedures [21]. These developments confirm the sustained relevance of deep learning architectures in clinical imaging workflows.

Beyond individual models, recent systematic reviews emphasize that vision–language models have revolutionized artificial intelligence by enabling scalable and multimodal learning in healthcare environments [22]. Furthermore, comprehensive adaptation frameworks have been proposed to tailor general-purpose VLMs specifically to medical applications, ensuring domain-specific robustness and interpretability [23].

Overall, the convergence of multimodal VLM architectures and automated analysis systems marks a transformative phase in medical image analysis, with promising implications for diagnosis, monitoring, and personalized treatment strategies.

However, they often require large annotated datasets and may suffer from limited interpretability and reduced generalization when faced with heterogeneous imaging protocols or acquisition conditions.

Motivated by these considerations, this work proposes a novel optimized handcrafted feature extraction framework for MRI-based Multiple Sclerosis (MS) diagnosis and assessment. The major aim of this study is to develop a robust, efficient, and discriminative feature extraction framework based on Gradient-enhanced Decimal Descriptor Patterns (DDP) for accurate multiple sclerosis (MS) detection and progression assessment using multi-modal MRI (FLAIR, T1, and T2). Specifically, this work seeks to enhance conventional handcrafted texture descriptors by incorporating local gradient information to better capture complex lesion characteristics, thereby improving classification performance across different disease stages.

The extracted handcrafted features are assessed using multiple classical machine learning classifiers, including Support Vector Machines (SVM) [24], Linear Discriminant Analysis (LDA) [25], k-Nearest Neighbors (KNN) [26], and Linear Regression [27]. To provide a comprehensive evaluation, the proposed handcrafted approach is systematically compared with a deep learning-based vision–language model, allowing an in-depth analysis of the respective strengths and limitations of handcrafted and deep feature representations in terms of diagnostic performance, robustness, and generalization.

The framework is validated using two complementary datasets: simulated T1/T2 MRI volumes from the BrainWeb dataset under varying imaging conditions [28], and a real-world FLAIR MRI dataset [29]. This dual validation strategy provides a comprehensive evaluation of robustness, generalizability, and practical applicability. Experimental results demonstrate that the proposed handcrafted feature-based approach achieves performance comparable to deep learning-based features while maintaining high stability across different MRI modalities and datasets, making it a reliable solution for automated MS diagnosis and progression monitoring.

The remainder of this paper is organized as follows. Section 2 presents the detailed methodology and algorithmic framework of our proposed approach. Section 3 presents experimental results and comparative evaluations, and Section 4 concludes the paper with final remarks and future research directions.

## 2. Methodology

This section details the complete processing pipeline designed for multiple sclerosis analysis, with particular emphasis on the feature extraction stage. The proposed Gradient-DDP method is investigated as an alternative to VLM-based feature representations and is comparatively evaluated using standard machine learning classifiers to assess its effectiveness for MS detection and progression.

**Figure 1:**
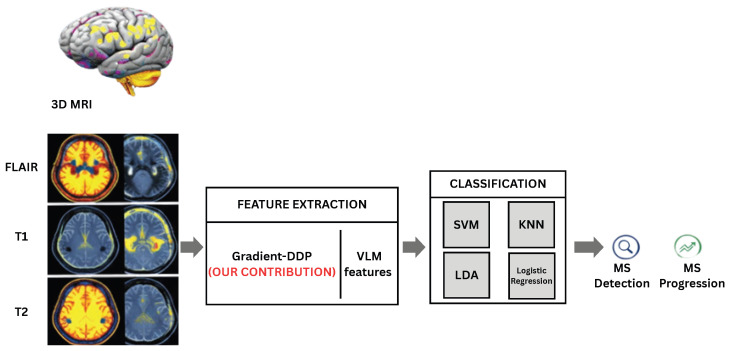
Overview of the proposed Gradient-DDP and VLM-based MS analysis pipeline.

Figure 1 provides an overview of the complete processing pipeline, including multi-modal MRI inputs, feature extraction strategies, classification models, and the final MS analysis tasks.

### 2.1. Feature Extraction

In this section, we present the proposed feature extraction approach. Since our contribution is based on DDP, we first introduce and explain the DDP method, to move then to explain our proposed feature extraction method.

#### 2.1.1. Decimal Descriptor Pattern (DDP)

In texture analysis, the Decimal Descriptor Pattern (DDP) has proven to be an effective method for characterizing image patterns [3,11,12,24]. DDP encodes the relationship between a pixel and its neighbors based on their intensity values, producing a compact feature vector that represents the texture of the image. For each pixel, DDP computes statistics such as the mean, maximum, and minimum within a 3×3 neighborhood, and concatenates these measurements across the image to form an initial feature vector $V_{i}$. This vector is then normalized and mapped into discrete codes ranging from 0 to 10, based on the minimum, maximum, and average values of $V_{i}$, resulting in a final coded feature vector $V_{f}$ that captures the essential texture information.

For each extracted feature vector $b={b_{i}}_{i=1}^{N}$, we first compute the local statistics:

(1) $$b_{\min}=\min\left(b\right),\;b_{\max}=\max\left(b\right),\;\mu =\frac{1}{N}\sum_{i=1}^{N}b_{i}$$

Each feature $b_{i}$ is then mapped to one of 11 discrete levels (0–10) according to a deterministic, rule-based scheme based on these statistics:

(2) $$Q\left(b_{i}\right)=\left\{\begin{array}{cc} 0 & b_{i}=b_{\min}\\ 1 & b_{i}=b_{\max}\\ 2 & b_{\min}<b_{i}\leq \left(\mu +b_{\min}\right)/4\\ 3 & \left(\mu +b_{\min}\right)/4<b_{i}\leq \left(\mu +b_{\min}\right)/2\\ 4 & \left(\mu +b_{\min}\right)/2<b_{i}\leq 3\left(\mu +b_{\min}\right)/4\\ 5 & 3\left(\mu +b_{\min}\right)/4<b_{i}<\mu \\ 6 & b_{i}=\mu \\ 7 & \mu <b_{i}\leq \left(\mu +b_{\max}\right)/4\\ 8 & \left(\mu +b_{\max}\right)/4<b_{i}\leq \left(\mu +b_{\max}\right)/2\\ 9 & \left(\mu +b_{\max}\right)/2<b_{i}\leq 3\left(\mu +b_{\max}\right)/4\\ 10 & 3\left(\mu +b_{\max}\right)/4<b_{i}<b_{\max} \end{array}\right.$$

#### 2.1.2. Proposed Gradient DDP-Based Feature Extraction Method

The main contribution of the proposed approach is the introduction of a gradient-driven local descriptor that replaces conventional intensity-based statistics in the DDP framework. Instead of summarizing each image block using minimum, maximum, and average pixel values, the proposed method characterizes local regions through spatial intensity variations, which provide a more informative representation of underlying structures.

By relying on gradient information, the descriptor becomes inherently sensitive to local transitions within the image. The gradient magnitude captures the strength of intensity changes, revealing boundaries and fine structural details, while the gradient direction encodes the geometric orientation of these transitions. This shift from absolute intensity values to variation-based cues allows the descriptor to emphasize structural patterns that may remain unnoticed when using only basic intensity summaries.

This strategy is particularly relevant for medical imaging applications such as magnetic resonance imaging (MRI) in multiple sclerosis (MS) analysis. MS lesions often exhibit subtle or heterogeneous contrast, making them difficult to detect using descriptors based solely on pixel intensity statistics. Gradient-based DDP features enhance the visibility of lesion contours and textural irregularities, leading to improved discrimination between pathological and normal tissue. Moreover, because gradients are less affected by uniform intensity shifts, the resulting representation is more robust to acquisition variability and noise.

**Figure 2:**
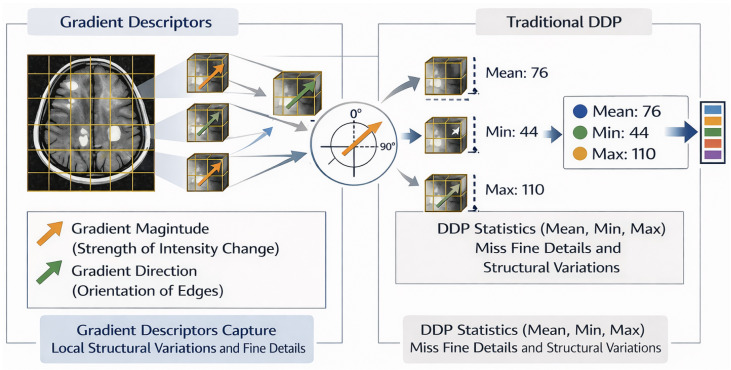
Gradient descriptor capturing local intensity changes and edge directions in MRI blocks.

Overall, replacing traditional DDP statistics with gradient-derived measures enables the construction of a richer and more discriminative feature space, better suited for machine learning-based classification of complex medical images (Figure 2).

The image gradient is defined as

(3) $$\nabla I=\left[\frac{\partial I}{\partial x},\frac{\partial I}{\partial y}\right]=\left[G_{x},G_{y}\right]$$

where I(x,y) denotes the image intensity function; $\frac{\partial I}{\partial x}$ and $\frac{\partial I}{\partial y}$ represent the first-order partial derivatives along the horizontal (*x*) and vertical (*y*) spatial directions, respectively. $G_{x}$ and $G_{y}$ correspond to the gradient components in the *x* and *y* directions, and ∇I denotes the gradient vector of the image.

As shown in Equation (3), the gradient vector describes the spatial rate of intensity variation in the image.

The gradient contains two main components derived from Equation (3):

- Magnitude:
  (4) $$G_{\mathrm{mag}}=\sqrt{G_{x}^{2}+G_{y}^{2}}$$
  where $G_{\mathrm{mag}}$ denotes the gradient magnitude, representing the strength of intensity variation at each pixel location. $G_{x}$ and $G_{y}$ are the horizontal and vertical gradient components defined in Equation (3). This measure highlights edges, boundaries, and regions of high contrast in the image.
- Direction (Orientation):
  (5) $$G_{\mathrm{dir}}=\arctan2\left(G_{y},G_{x}\right)$$
  where $G_{\mathrm{dir}}$ represents the gradient orientation (in radians), and arctan2(·) is the four-quadrant inverse tangent function that computes the angle between the positive *x*-axis and the vector $\left(G_{x},G_{y}\right)$. This component indicates the direction of maximum intensity change, revealing the orientation of edges or structural patterns.

Gradients are fundamental in image analysis because they capture local variations rather than absolute intensity values. This property makes them highly useful for tasks such as edge detection, texture characterization, and feature extraction, allowing machine learning models to identify structural patterns and subtle differences between classes, which is particularly important in medical imaging applications. Figure 2 illustrates the proposed approach which introduces a gradient descriptor that replaces traditional intensity-based statistics in the DDP framework. By capturing local intensity variations through gradient magnitude and direction, it emphasizes boundaries and fine structural patterns. We used the standard numerical gradient as implemented in numpy.gradient, which computes partial derivatives along the *x* and *y* directions.

#### 2.1.3. Detailed Methodology and Algorithmic Framework

**Figure 3:**
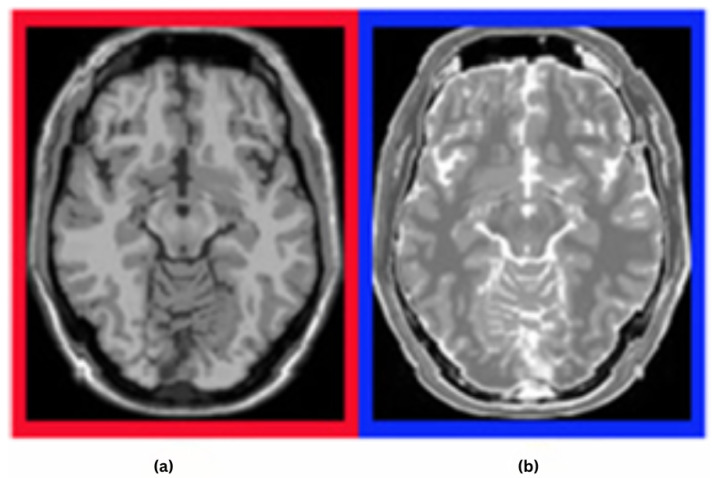
Examples of MRI sequences of an MS brain: (**a**) T1- and (**b**) T2-weighted images [3].

Our study focuses on two main objectives: the detection of multiple sclerosis (MS) and the monitoring of its progression using flair, T1- and T2-weighted MRI. Examples of MRI sequences of an MS brain are illustrated in Figure 3a (a T1-weighted image, which provides detailed anatomical structure) and Figure 3b (a T2-weighted image, which highlights lesions and fluid abnormalities).

**Figure 4:**
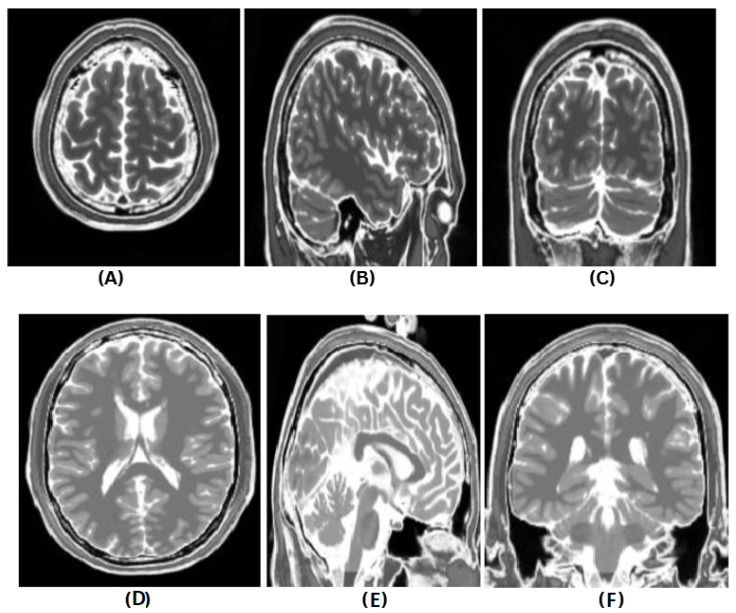
Example of T2-weighted MRI slices from the BrainWeb dataset illustrating healthy brain anatomy and multiple sclerosis (MS) lesions across different anatomical planes. (**A**) Axial (transversal) view of a healthy brain; (**B**) sagittal view of a healthy brain; (**C**) coronal view of a healthy brain. (**D**) Axial (transversal) view of a brain with MS lesions; (**E**) sagittal view of a brain with MS lesions; (**F**) coronal view of a brain with MS lesions.

For the first objective, we aim to accurately identify whether a subject is affected by MS or not using advanced machine learning techniques applied to MRI data as illustrated in Figure 4. The images correspond to different anatomical planes, where (A)–(C) represent axial (transversal), sagittal, and coronal views of a healthy brain, and (D)–(F) show the same views for a brain with MS lesions.

The second objective involves assessing the severity and evolution of the disease over time, allowing for classification of MS into different stages. These categories are defined based on quantitative and radiological criteria derived from longitudinal MRI analysis, including lesion load (number and volume of lesions) and spatial distribution.

**Figure 5:**
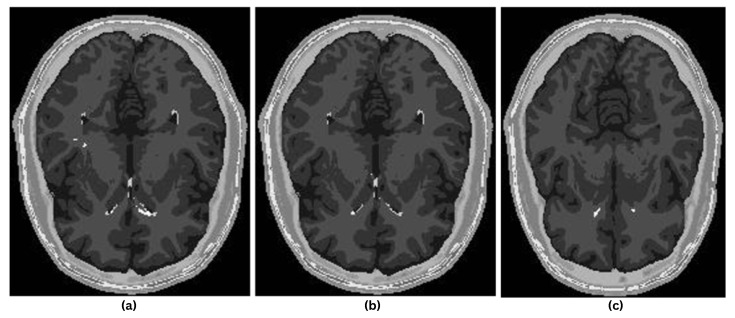
MRI Examples Illustrating Disease Progression Levels: (**a**) Severe, (**b**) Moderate, (**c**) Mild.

Figure 5 shows representative MRI slices illustrating three stages of disease progression. From right to left, the images correspond to mild, moderate, and severe lesion burden. The lesion size visibly increases across the stages, reflecting the progression and severity of the disease.

In this study, both the handcrafted descriptors (Gradient-DDP) and the VLM-based features (CLIP) are extracted from 2D slices independently. This slice-based approach was chosen to reduce computational complexity while enabling efficient analysis of large 3D MRI volumes. Moreover, many previous studies on multiple sclerosis lesion analysis have successfully applied 2D slice-based methods with strong performance. To incorporate 3D information, features extracted from individual slices are aggregated across the volume, allowing the model to capture volumetric patterns implicitly without directly processing full 3D data. This strategy provides a practical balance between computational efficiency and effective modeling of spatial lesion characteristics.

In Algorithm 1 we detail the binary classification of Multiple Sclerosis (MS) using MRI data.

**Algorithm 1:** Binary MS Classification Using Gradient-DDP Features with Subject-Wise Stratified Splitting

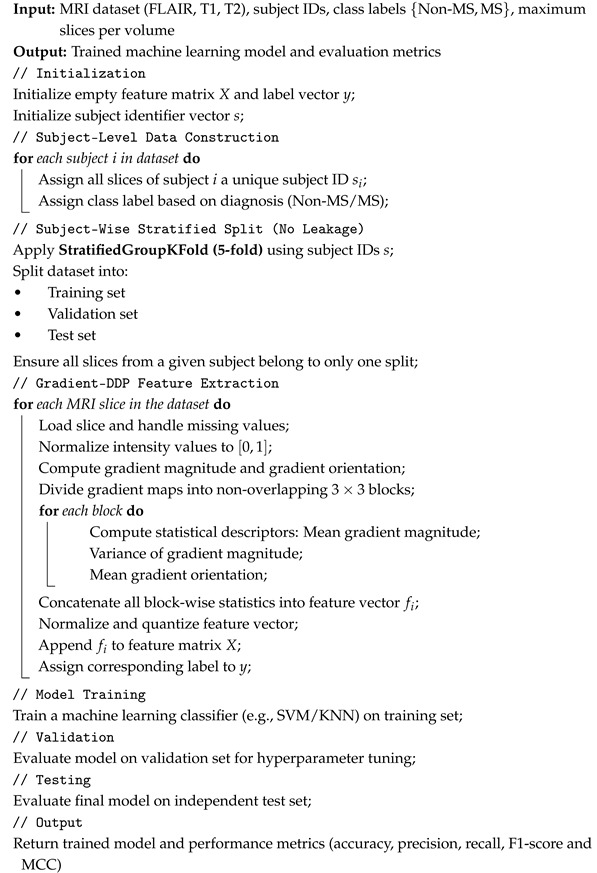



    The second study addresses the multi-class classification of Multiple Sclerosis (MS). Once the MS is detected in an MRI, we move to study the disease progression by distinguishing between mild, moderate, and severe forms. To achieve that, as demonstrated in Algorithm 2, we employ the proposed DDP gradient-based method to extract detailed features from MRI images, which are then processed by a multi-class machine learning model to accurately identify disease severity and facilitate monitoring of MS progression.

**Algorithm 2:** Multi-Class MS Severity Classification Using Gradient-DDP Features with Subject-Wise Stratified Splitting

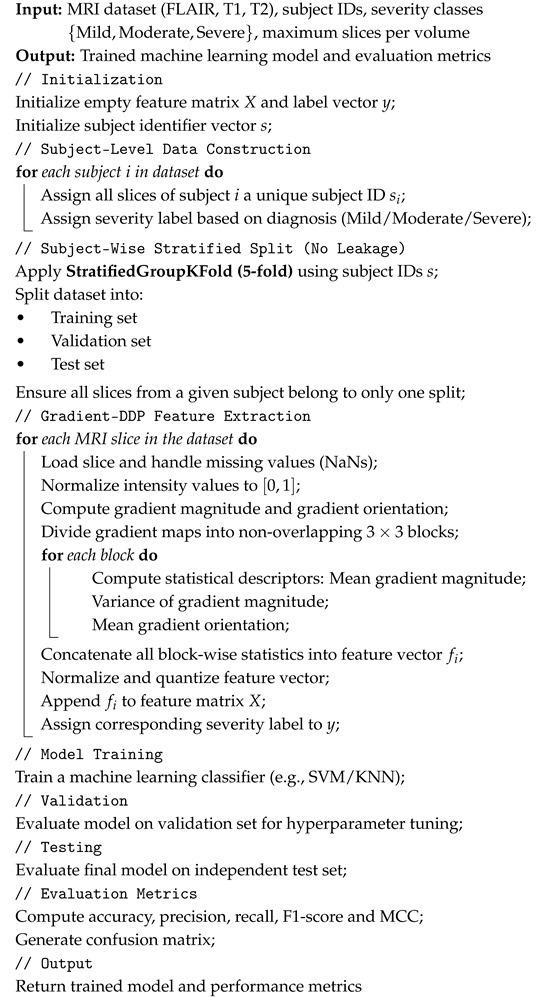



Synthetic elliptical lesions are introduced to address the limited availability of annotated data representing different Multiple Sclerosis (MS) severity levels. Due to the scarcity and imbalance of real-world datasets, especially for progression stages, synthetic augmentation is a commonly adopted strategy in medical imaging studies. Moreover, the generated lesions are designed to approximate realistic variations in size, shape, and distribution observed in MS, allowing the model to learn more diverse patterns.

For comparison purposes between handcrafted feature extraction methods and deep feature extraction approaches, we selected vision–language model (Clip) as a deep technique to perform feature extraction and compare it with our proposed approach. For this reason, VLMs are discussed in the following section.

#### 2.1.4. VLM for Feature Extraction

**Figure 6:**
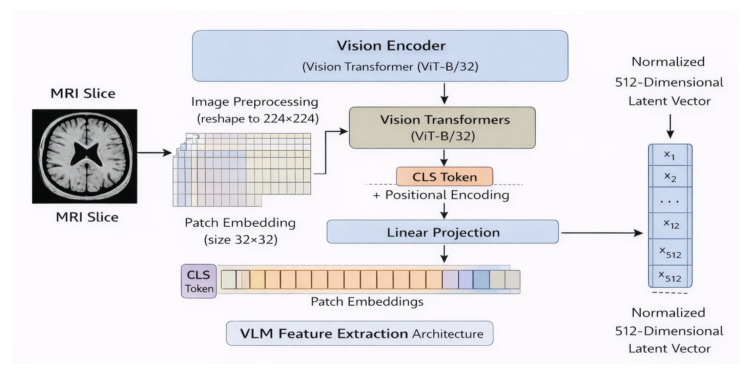
Architecture of the CLIP Visual Encoder (ViT-B/32) for MRI Feature Extraction.

A vision–language model (VLM) extracts information from an image by first processing it through a vision encoder, which analyzes visual content and produces a high-dimensional feature vector. This vector captures important patterns such as shapes, textures, colors, and spatial relationships within the image. The features are often normalized to ensure consistency between different images. These extracted features can then be used as input for downstream tasks, such as classification, detection, or segmentation, allowing machine learning models to make predictions based on the rich representations provided by the VLM. Figure 6 illustrates the used architecture.

In this study, we employ the CLIP (Contrastive Language–Image Pretraining) model from OpenAI by utilizing only its visual encoder as a feature extraction backbone. Specifically, the ViT-B/32 variant is adopted, where each MRI slice is resized to 224 × 224 pixels, partitioned into 32 × 32 patches, and converted into patch embeddings augmented with positional encodings. These embeddings are processed through multiple Transformer layers with multi-head self-attention to capture both local structural patterns and global spatial relationships. A final linear projection maps the global representation (CLS token) into a 512-dimensional latent vector, which is L2-normalized within the shared embedding space originally learned through contrastive image–text pretraining.

The choice of ViT-B/32 was primarily motivated by a trade-off between computational efficiency and model capacity. Compared to larger variants such as ViT-L/16, ViT-B/32 requires significantly fewer computational resources and is less prone to overfitting, which is particularly important given the relatively limited size of our dataset [30,31,32].

Additionally, ViT-B/32 operates with larger patch sizes, resulting in a lower number of tokens and reduced memory consumption, making it more suitable for our experimental setup. While smaller patch variants such as ViT-B/16 can capture finer spatial details, they also increase model complexity and computational cost, which may not translate into improved performance in low-data regimes.

In our framework, each MRI slice is normalized using min–max scaling to enhance image contrast. The slices are then converted to three-channel (RGB) images and processed using CLIP’s standard preprocessing pipeline via the CLIPProcessor. This pipeline includes resizing slices, center cropping, conversion to tensor format, and normalization using CLIP’s pretrained mean and standard deviation. Although the initial min–max normalization is an additional step specific to MRI data, it does not interfere with CLIP’s standard preprocessing and does not introduce any bias or data leakage. This approach ensures that the inputs are compatible with CLIP’s visual encoder while preserving the intensity characteristics of medical images, facilitating effective feature extraction for downstream tasks. The resulting normalized embeddings are then provided to supervised classifiers (SVM, KNN, and Logistic Regression) and are used with standard scikit-learn defaults for multiple sclerosis detection and severity assessment, forming the basis of the diagnostic framework described in the next section.

### 2.2. MS Diagnosis

Once feature extraction is completed, the framework proceeds to the Multiple Sclerosis (MS) diagnostic stage. In other words, the system studied the progression level of MS (mild, moderate or severe). This analysis first requires accurate disease detection; therefore, the extracted feature representations are fed into binary supervised classifiers, including SVM, KNN, Logistic Regression, and LDA. In case MS is detected, the features will be fed to multiclass classifiers to make a decision about the severity of the disease.

#### 2.2.1. Support Vector Machines (SVM)

Support Vector Machines (SVM) are supervised classification algorithms that aim to construct an optimal separating boundary between different categories of data. The core concept of SVM is to identify a decision surface, known as a hyperplane, in a high-dimensional feature space that maximizes the distance between the boundary and the nearest training samples from each class, referred to as support vectors. Increasing this margin improves the model’s ability to generalize and enhances its robustness when classifying unseen samples [24].

#### 2.2.2. Linear Discriminant Analysis (LDA)

Linear Discriminant Analysis (LDA) is a supervised machine learning technique used for classification and dimensionality reduction [24,25]. Its main principle is to project high-dimensional data onto a lower-dimensional space in a way that maximizes the separation between classes while minimizing the variance within each class. By doing so, LDA enhances the discriminative power of the features, making it easier to distinguish between categories. Additionally, it provides a linear combination of input features, which offers interpretability, reduces computational complexity, and can improve the performance of subsequent classifiers, especially when dealing with moderate-sized datasets.

#### 2.2.3. K-Nearest Neighbors (KNN)

K-Nearest Neighbors (KNN) is a supervised machine learning algorithm used for classification and regression [24,26]. It predicts the label or value of a new data point based on the majority class or average of its k closest neighbors in the feature space. KNN is simple, intuitive, and non-parametric, requiring no explicit training model, and it naturally adapts to complex data distributions. Its performance depends on the choice of k and the distance metric, making it particularly effective for datasets where similar instances are expected to have similar outcomes.

#### 2.2.4. Logistic Regression

Logistic Regression is a supervised machine learning technique used to predict a continuous outcome based on one or more input features [27]. It models the relationship between the dependent variable and the predictors as a straight line (or hyperplane in multiple dimensions), estimating coefficients that best fit the data by minimizing prediction errors. Logistic regression is valued for its simplicity, interpretability, and efficiency, allowing one to understand the influence of each feature while making predictions or analyzing trends in the data.

## 3. Experiments

This section describes the experimental setup established to assess the effectiveness of the proposed framework for Multiple Sclerosis detection and severity classification. The evaluation relies on two distinct MRI datasets to examine the robustness and generalization capability of the approach. Moreover, a comparative study is conducted between two feature extraction strategies to determine their impact on classification performance. The resulting feature representations are evaluated using four supervised learning algorithms: SVM, KNN, Logistic Regression, and LDA. A K-Nearest Neighbors (KNN) classifier was used with k = 3, employing distance-based weighting, and the Minkowski distance metric (*p* = 2, equivalent to Euclidean distance) to improve classification sensitivity to closer neighbors. An SVM classifier with GridSearch optimization was applied on T2-weighted MRI data, yielding the best hyperparameters—C = 10, γ = ‘scale’, and an RBF kernel—indicating optimal nonlinear separation performance. LDA was implemented using its default configuration, with the SVD solver, no shrinkage, and without prior probability tuning, ensuring a stable linear projection for class separation. Linear Regression was applied using its default parameters, with no regularization (ordinary least squares), no intercept constraint modification, and relying on direct minimization of the squared error between predicted and target class labels. The experimental results are presented and thoroughly analyzed to compare the effectiveness of the different methods.

### 3.1. Dataset Description

The proposed framework is evaluated on two complementary MRI datasets comprising three imaging contrasts, T1, T2, and FLAIR, in order to assess its stability and consistency across different acquisition modalities. Two complementary datasets were employed to assess the robustness and generalizability of the proposed framework: BrainWeb and MS NonMS dataset.

#### 3.1.1. BrainWeb Dataset

The BrainWeb dataset consists of MRI volumes with dimensions of 181 × 217 × 181 voxels, offering a highly detailed spatial representation of the brain’s anatomy [28]. It encompasses distinct tissue types including gray matter, white matter, and cerebrospinal fluid (CSF), each paired with ground truth segmentations, enabling precise evaluation and validation of feature extraction and classification methods. BrainWeb enables the adjustment of key imaging parameters including slice thickness, signal-to-noise ratio (SNR), and intensity non-uniformity, allowing the simulation of diverse MRI acquisition conditions.

The Simulated Brain Database (SBD) contains realistic 3D MRI volumes for normal and multiple sclerosis (MS) brain models, including T1-, T2-, and proton-density (PD)-weighted sequences. The T1-weighted scans used to generate the high-resolution phantom were based on 27 averaged gradient echo acquisitions with the following parameters:

- TR = 18 ms;
- TE = 10 ms;
- Flip angle = 30°;
- Voxel size = 1 mm^3^ isotropic.

This covers the entire brain from the top of the scalp to the foramen magnum. These high-resolution scans preserve fine anatomical details such as subthalamic nuclei, claustrum, gray matter bridges, and vessels within the lentiform nucleus.

The T1- and T2-weighted MRI volumes were simulated based on the BrainWeb database, which provides 3D volumes with 1 mm^3^ isotropic resolution and default images without noise or intensity non-uniformity (INU). To mimic realistic MR acquisitions, controlled levels of noise, slice thickness, and INU were applied. These parameters ensure that the SBD provides a precise reference for evaluating image processing algorithms while reflecting realistic acquisition conditions.

In addition, the dataset supports synthetic lesion generation, which is particularly valuable for assessing the performance of automated lesion detection and segmentation algorithms [28].

#### 3.1.2. Real-World FLAIR MRI Dataset

The real-world FLAIR MRI data used for MS versus non-MS classification included 60 training and 110 test cases acquired from three centers (Netherlands and Singapore) using five different scanners (Philips, Siemens, GE). Each subject had 2D/3D FLAIR images, with manual white matter hyperintensity (WMH) masks provided for training. All images were bias-field-corrected and aligned, and WMH annotations were reviewed by expert raters according to STRIVE criteria [29].

### 3.2. Experiment Details

The study employed a structured framework for MRI-based lesion classification. Feature extraction was performed using a gradient Decimal Descriptor Pattern on 3 × 3 patches from each slice. Synthetic lesions with varying severity levels were added to the MRI slices, and the resulting values were aggregated into normalized histograms with 16 bins. The BrainWeb dataset comprised 120 slices per subject, and the real-world FLAIR MRI dataset comprised 50 slices per subject across the specified subjects. All slices originating from a given subject are now assigned exclusively to either the training, validation, or test set, with no overlap across subsets. This strict partitioning was enforced using subject identifiers within a StratifiedGroupKFold framework, ensuring that no information from a single subject was shared across different splits and thereby effectively eliminating any risk of data leakage. Furthermore, a dedicated validation set was explicitly introduced, and the dataset was partitioned using a 5-fold cross-validation strategy to provide a more stable and reliable evaluation protocol, improving the robustness and generalizability of the experimental results. Table 1 presents the dataset distribution used for the binary multiple sclerosis (MS) detection task across the BrainWeb and FLAIR datasets, including the number of subjects, slices, and the distribution of non-MS and MS classes across the training, validation, and test sets.

Table 2 summarizes the dataset distribution used for the three-class multiple sclerosis (MS) progression classification task across both BrainWeb and FLAIR datasets, detailing the number of subjects, slices, and class-wise distribution for the training, validation, and testing splits.

The proposed algorithm captures the main steps of the MS progression and assessment pipeline. First, gradient-based DDP feature extraction is performed on each 2D MRI slice. This step involves computing the horizontal and vertical gradients, calculating the magnitude and direction of the gradient, and dividing the image into small blocks to compute local statistics such as mean, variance, and mean gradient direction. These local measures are then combined into a feature vector for each slice, normalized, and encoded into discrete levels to effectively capture textural and edge-related information. Next, a data set is constructed by aggregating the feature vectors of all slices and subjects along with their corresponding severity labels. Finally, a machine learning model is trained on this data set to classify the severity of MS progression, and its performance is evaluated using standard metrics such as accuracy, precision, recall, F1-score, and confusion matrix.

To ensure a fair comparison between handcrafted feature extraction methods and deep learning-based representations, a vision–language model (VLM) is employed as a feature extractor and coupled with conventional machine learning classifiers. This strategy allows us to assess the discriminative power of features learned by the VLM in contrast to those derived from the proposed handcrafted approach.

### 3.3. Results and Discussion

Building on the experimental setup and dataset overview, the following subsections present a detailed analysis of the results, highlighting classifier performance, which are detailed in the following section for MS detection and MS progression assessment.

#### 3.3.1. MS Identification

Table 3 summarizes the performance of binary MS classification using different classifiers, feature extraction strategies, and MRI modalities.

To further evaluate the classification behavior of each algorithm, Table 4 presents the confusion matrices obtained from binary classification of Multiple Sclerosis (MS) versus non-MS subjects using different machine learning models applied to Flair, T1- and T2-weighted MRI data.

Each matrix displays the distribution of correctly and incorrectly classified samples, illustrating the models’ ability to separate MS lesions from non-MS regions.

In addition, as shown in Table 5, we computed the Matthews Correlation Coefficient (MCC) across MRI modalities, reporting both the mean and standard deviation, to provide a robust and comprehensive assessment of model performance and stability.

##### Discussion: MS Detection

The performance of the proposed pipeline for binary MS detection (MS vs. non-MS) is summarized in Table 3, while the corresponding confusion matrices are reported in Table 4. Overall, the results demonstrate that both the choice of MRI modality and the feature extraction method significantly impact classification performance.

Using Gradient-DDP features, all classifiers achieve high and consistent performance across modalities, with the best results obtained on FLAIR images, where accuracy, precision, recall, and F1-score reach 0.99 for all models (SVM, KNN, LDA, and Logistic Regression). This near-perfect performance is confirmed by the confusion matrices, which show almost zero false negatives (e.g., SVM: $\left[\begin{array}{ccc} 1522 & 28\;0 & 900 \end{array}\right]$), indicating excellent sensitivity to MS lesions.

For T2-weighted MRI, Gradient-DDP also yields strong performance, particularly with SVM (0.98 accuracy, 0.98 F1-score) and LDA (0.96 accuracy), while Logistic Regression achieves 0.93 accuracy. The confusion matrices indicate low misclassification rates (e.g., SVM: $\left[\begin{array}{ccc} 221 & 7\;3 & 249 \end{array}\right]$), confirming good separability between MS and non-MS samples.

In contrast, T1-weighted MRI shows comparatively lower performance, with accuracies ranging from 0.90 (SVM and Logistic Regression) to 0.97 (LDA) using Gradient-DDP features. This is reflected in slightly higher false negative rates (e.g., SVM: $\left[\begin{array}{ccc} 238 & 1\;15 & 226 \end{array}\right]$), which can be explained by the reduced contrast of MS lesions in T1 images.

When using VLM-based features, performance is consistently lower and more variable across all modalities. For instance, SVM achieves 0.94 accuracy on T1 and T2, but drops significantly to 0.85 on FLAIR. Similarly, KNN and Logistic Regression show accuracies around 0.80–0.93, depending on the modality. The degradation is particularly pronounced in FLAIR, where confusion matrices reveal a substantial increase in both false positives and false negatives (e.g., SVM: $\left[\begin{array}{ccc} 1377 & 173\;204 & 696 \end{array}\right]$).

Across classifiers, SVM and LDA exhibit the most stable and highest performance when combined with Gradient-DDP features, suggesting that the extracted features provide strong class separability. KNN shows slightly more variability, likely due to its sensitivity to local data distribution, while Logistic Regression maintains competitive but slightly lower performance.

In summary, the results indicate that FLAIR combined with Gradient-DDP features provides the most accurate and robust solution for MS detection, followed by T2 and T1 modalities. Moreover, gradient-based handcrafted features consistently outperform VLM representations, highlighting the importance of domain-specific feature extraction in medical imaging tasks.

As shown in Table 5, the MCC analysis highlights near-perfect classification performance for FLAIR images when using Gradient-DDP features, with consistently high values across all classifiers (e.g., 0.981 for SVM, 0.979 for KNN, 0.968 for LDA, and 0.979 for Logistic Regression). These results confirm the strong discriminative capability of Gradient-DDP on FLAIR data, where lesion contrast is maximized.

For T2-weighted MRI, Table 5 shows that MCC values remain high with Gradient-DDP (e.g., 0.938 for SVM, 0.929 for KNN and LDA), indicating stable and reliable performance across models. In contrast, T1-weighted MRI exhibits slightly lower MCC values, particularly for KNN (0.729), although linear models such as LDA and Logistic Regression still achieve strong performance (around 0.93). This trend reflects the lower lesion visibility in T1 images.

Furthermore, Table 5 clearly demonstrates that Gradient-DDP consistently outperforms VLM-based features in both performance and stability. For instance, SVM with Gradient-DDP achieves a mean MCC of 0.951 ± 0.021, compared to 0.813 ± 0.085 for VLM. Similarly, LDA and Logistic Regression show lower mean MCC values and higher variability with VLM (e.g., 0.721 ± 0.122 and 0.708 ± 0.110, respectively). This degradation is particularly evident in FLAIR, where VLM-based MCC drops to approximately 0.56–0.69.

Overall, these results, as summarized in Table 5, emphasize the robustness, stability, and superior discriminative power of Gradient-DDP features, especially when combined with FLAIR imaging. In contrast, VLM-based representations show reduced performance and higher variability, likely due to their limited adaptation to domain-specific medical imaging characteristics.

#### 3.3.2. MS Progression Classification

Table 6 shows that multi-class classification of MS severity is strongly influenced by the imaging modality and the feature representation used.

Table 7 presents the confusion matrices for the four classification models applied to three-class Multiple Sclerosis (MS) diagnosis (MS mild, MS moderate, MS severe). The evaluation was performed on both T1- and T2-weighted MRI modalities. Each confusion matrix summarizes the number of correctly and incorrectly classified samples, providing a detailed view of model performance across all severity levels.

Additionally, Table 8 reports the Matthews Correlation Coefficient (MCC) for multi-class MS severity classification across MRI modalities, along with the corresponding mean and standard deviation, to provide a more robust and comprehensive evaluation of model performance and stability.

##### Discussion: MS Progression

The results presented in Table 6 demonstrate notable differences in classification performance across MRI modalities, models, and feature extraction strategies for three-class MS progression.

Overall, Gradient-DDP features consistently outperform VLM-based features, particularly for T1 and FLAIR modalities. For instance, using SVM, Gradient-DDP achieves 96% accuracy on T2, compared to 80% on T1 and 84% on FLAIR, while VLM shows a significant drop on FLAIR (62% accuracy) and T1 (66% accuracy).

T2-weighted MRI consistently yields the best results across all models and feature extraction methods. Gradient-DDP achieves 96% accuracy with SVM and Logistic Regression, while VLM also performs strongly (96–97% accuracy with SVM and KNN). This suggests that T2 images provide clearer contrast between MS severity levels, making them highly discriminative regardless of the feature extraction method.

In contrast, T1-weighted MRI shows more variability. Gradient-DDP achieves moderate performance (80–81% accuracy with SVM and LDA), while VLM performance ranges widely—from 49% (Logistic Regression) to 95% (LDA). This instability indicates that VLM features may not consistently capture relevant pathological patterns in T1 images, whereas Gradient-DDP provides more stable and interpretable descriptors.

For FLAIR MRI, Gradient-DDP again demonstrates strong robustness, achieving up to 84% accuracy (SVM), compared to significantly lower results for VLM (as low as 51–62% for SVM and LDA). This represents a relative improvement of more than 20–30 percentage points, highlighting the advantage of gradient-based features in capturing lesion-specific information in FLAIR images.

The superiority of Gradient-DDP can be explained by its direct modeling of local structural variations, particularly gradient magnitude and orientation, which are closely related to lesion boundaries and texture irregularities. MS lesions typically manifest as intensity discontinuities and structural deformations, which are effectively captured by gradient-based descriptors.

In contrast, VLM (CLIP-based) features are high-level semantic embeddings, originally trained on natural images. While they perform well on T2 images (where lesion contrast is strong), they struggle with FLAIR and T1 modalities, likely due to

- Domain mismatch between natural images and medical MRI;

- Reduced sensitivity to fine-grained texture variations;

- Loss of spatial detail during embedding extraction.

The confusion matrices (Table 7) further support these observations. With Gradient-DDP, T2 modality shows minimal confusion, with most samples correctly classified across all severity levels.

FLAIR modality exhibits moderate confusion, mainly between adjacent classes (mild vs. moderate), but still maintains strong diagonal dominance. T1 modality shows higher confusion, especially for moderate cases, explaining the lower accuracy.

For VLM, FLAIR confusion matrices reveal significant misclassification, with widespread off-diagonal values, confirming the drop in performance. T1 results are inconsistent, with some models (e.g., LDA) performing well, while others fail to generalize.

These results clearly indicate that Gradient-DDP provides a more robust and modality-consistent feature representation, particularly for clinically relevant modalities such as FLAIR and T1. While VLM features can achieve competitive performance on T2 images, their instability across other modalities limits their reliability for MS progression classification.

The extended MCC analysis confirms that SVM combined with Gradient-DDP features achieves the best overall performance in multi-class MS severity classification, reaching a mean MCC of 0.81. This represents an improvement of approximately +45% compared to its VLM-based counterpart (0.56). More broadly, Gradient-DDP features consistently provide more stable and reliable results across MRI modalities, reducing variability by up to 50% in terms of standard deviation compared to VLM-based features. In terms of modalities, T2-weighted images remain the most discriminative, achieving MCC values as high as 0.95–0.96, which corresponds to an improvement of nearly +30–50% over T1 and FLAIR in several configurations. In contrast, FLAIR exhibits notable limitations for severity classification, with MCC drops reaching up to −50% to −70% in certain VLM-based settings. These findings highlight the superiority of Gradient-DDP features in terms of both performance and robustness, as well as the critical role of T2 imaging in accurately assessing MS progression.

Although the method is robust and interpretable, it may not fully capture all subtle lesion variations. Future work will focus on evaluating the proposed approach on larger, multi-center cohorts to further assess its generalizability and clinical applicability.

#### 3.3.3. Comparative Results of Multiple Sclerosis (MS) Detection and Progression Studies

This section highlights the performance of various algorithms, MRI modalities, and experimental conditions to evaluate the robustness and effectiveness of the proposed approach. The comparative results in Table 9 indicate that the proposed Gradient-DDP approach achieves performance that is fully consistent with, and in several cases comparable to, existing methods reported in the literature. For MS detection, our method reaches 90% accuracy on T1, 98% on T2, and 99% on FLAIR, which aligns closely with prior works such as [3,33], where accuracies range from 93% to nearly 100% depending on modality and experimental conditions. Similarly, for MS progression, the obtained results (80% on T1 and 96% on T2) are competitive with earlier DDP-based approaches, including DDP-NOP, which reported around 93.9% on T1.

These findings confirm that Gradient-DDP remains a robust and reliable feature extraction strategy, capable of capturing discriminative structural patterns in MRI data. Moreover, the strong performance across multiple modalities—particularly the high accuracy on T2 and FLAIR—suggests that gradient-based descriptors effectively encode lesion-related information, making them well-suited for both detection and progression tasks. Overall, the proposed approach not only corroborates previous results but also demonstrates its ability to generalize across different MRI modalities while maintaining high classification performance.

## 4. Conclusions

This study addresses the challenges of automated Multiple Sclerosis (MS) diagnosis by introducing an optimized feature extraction framework tailored for complex 3D brain data. The research focused specifically on two critical clinical objectives: the detection of MS and the assessment of disease progression. By enhancing Decimal Descriptor Patterns with local gradient information (Gradient-DDP), we developed a 3D texture representation that is highly expressive across multiple MRI modalities, including FLAIR, T1-, and T2-weighted images.

The results demonstrate that the proposed handcrafted feature extraction is comparable to features extracted via vision–language models (VLM) in terms of accuracy. However, Gradient-DDP offers distinct advantages in consistency and transparency, providing a clear and interpretable relationship between texture patterns and diagnostic outcomes. While VLM features excel when lesion patterns are clearly defined in T1 and FLAIR, the Gradient-DDP method provides superior robustness in more challenging modalities like T2, where deep representations can be less stable. 

## Figures and Tables

**Table 1:** Dataset distribution for MS detection (binary classification) across BrainWeb and FLAIR datasets.

Dataset	Split	Slices	Class 0 (Non-MS)	Class 1 (MS)	Subjects
BrainWeb	Train	1440	714	726	12
	Validation	480	236	244	4
	Test	480	250	230	4
FLAIR	Train	7850	6050	1800	157
	Validation	1950	1550	400	39
	Test	2450	1550	900	49

**Table 2:** Dataset distribution for 3-class MS progression classification across BrainWeb and FLAIR datasets.

Dataset	Split	Subjects	Slices	Class 0	Class 1	Class 2
BrainWeb	Train	13	1560	506	508	546
	Validation	3	360	122	107	131
	Test	4	480	164	177	139
FLAIR	Train	196	9800	3256	3184	3360
	Validation	39	1950	651	755	544
	Test	49	2450	787	858	805
	Total	245	12250	4043	4042	4165

**Table 3:** Binary classification results (MS vs. non-MS) across different models, feature extraction methods, and MRI modalities.

Model	Feature Extraction Method	Modality	Precision	Recall	F1-Score	Accuracy
SVM	Gradient-DDP	T1	0.91	0.90	0.90	0.90
		T2	0.98	0.98	0.98	0.98
		Flair	0.99	0.99	0.99	0.99
	VLM	T1	0.97	0.90	0.93	0.94
		T2	0.97	0.90	0.93	0.94
		Flair	0.85	0.85	0.85	0.85
KNN	Gradient-DDP	T1	0.94	0.94	0.94	0.94
		T2	0.92	0.90	0.90	0.90
		Flair	0.99	0.99	0.99	0.99
	VLM	T1	0.91	0.89	0.90	0.91
		T2	0.97	0.89	0.93	0.93
		Flair	0.80	0.80	0.80	0.80
LDA	Gradient-DDP	T1	0.97	0.97	0.97	0.97
		T2	0.96	0.96	0.96	0.96
		Flair	0.99	0.99	0.99	0.99
	VLM	T1	0.82	0.96	0.88	0.88
		T2	1.00	0.85	0.92	0.93
		Flair	0.80	0.80	0.80	0.80
Logistic Reg	Gradient-DDP	T1	0.91	0.90	0.90	0.90
		T2	0.93	0.93	0.93	0.93
		Flair	0.99	0.99	0.99	0.99
	VLM	T1	0.82	0.96	0.88	0.88
		T2	0.92	0.91	0.91	0.91
		Flair	0.80	0.80	0.80	0.80

**Table 4:** Confusion matrices summarizing the classification performance of binary MS vs. non-MS prediction across different models, feature extraction methods, and MRI modalities.

Model	Feature Extraction Method	Modality	Confusion Matrix
SVM	Gradient-DDP	T1	$\left[\begin{array}{cc} 238 & 1\\ 15 & 226 \end{array}\right]$
		T2	$\left[\begin{array}{cc} 221 & 7\\ 3 & 249 \end{array}\right]$
		Flair	$\left[\begin{array}{cc} 1522 & 28\\ 0 & 900 \end{array}\right]$
	VLM	T1	$\left[\begin{array}{cc} 200 & 23\\ 7 & 250 \end{array}\right]$
		T2	$\left[\begin{array}{cc} 200 & 23\\ 7 & 250 \end{array}\right]$
		Flair	$\left[\begin{array}{cc} 1377 & 173\\ 204 & 696 \end{array}\right]$
KNN	Gradient-DDP	T1	$\left[\begin{array}{cc} 197 & 43\\ 22 & 218 \end{array}\right]$
		T2	$\left[\begin{array}{cc} 233 & 7\\ 10 & 230 \end{array}\right]$
		Flair	$\left[\begin{array}{cc} 1518 & 32\\ 0 & 900 \end{array}\right]$
	VLM	T1	$\left[\begin{array}{cc} 199 & 24\\ 20 & 237 \end{array}\right]$
		T2	$\left[\begin{array}{cc} 223 & 27\\ 6 & 224 \end{array}\right]$
		Flair	$\left[\begin{array}{cc} 1318 & 232\\ 261 & 639 \end{array}\right]$
LDA	Gradient-DDP	T1	$\left[\begin{array}{cc} 238 & 1\\ 15 & 226 \end{array}\right]$
		T2	$\left[\begin{array}{cc} 234 & 6\\ 11 & 229 \end{array}\right]$
		Flair	$\left[\begin{array}{cc} 1519 & 31\\ 4 & 896 \end{array}\right]$
	VLM	T1	$\left[\begin{array}{cc} 215 & 8\\ 48 & 209 \end{array}\right]$
		T2	$\left[\begin{array}{cc} 193 & 34\\ 0 & 253 \end{array}\right]$
		Flair	$\left[\begin{array}{cc} 1406 & 144\\ 336 & 564 \end{array}\right]$
Logistic Regression	Gradient-DDP	T1	$\left[\begin{array}{cc} 237 & 2\\ 14 & 227 \end{array}\right]$
		T2	$\left[\begin{array}{cc} 221 & 16\\ 20 & 223 \end{array}\right]$
		Flair	$\left[\begin{array}{cc} 2405 & 26\\ 2 & 1487 \end{array}\right]$
	VLM	T1	$\left[\begin{array}{cc} 215 & 8\\ 48 & 209 \end{array}\right]$
		T2	$\left[\begin{array}{cc} 203 & 44\\ 1 & 232 \end{array}\right]$
		Flair	$\left[\begin{array}{cc} 1409 & 141\\ 341 & 559 \end{array}\right]$

**Table 5:** Matthews Correlation Coefficient (MCC) summarizing the classification performance of binary MS vs. non-MS prediction across MRI modalities with mean and standard deviation.

Model	Feature Extraction	MCC (T1/T2/FLAIR) + Mean ± Std
SVM	Gradient-DDP	0.934/0.938/0.981 (0.951 ± 0.021)
SVM	VLM (CLIP)	0.873/0.873/0.693 (0.813 ± 0.085)
KNN	Gradient-DDP	0.729/0.929/0.979 (0.879 ± 0.107)
KNN	VLM (CLIP)	0.816/0.864/0.613 (0.764 ± 0.108)
LDA	Gradient-DDP	0.934/0.929/0.968 (0.944 ± 0.018)
LDA	VLM (CLIP)	0.748/0.856/0.559 (0.721 ± 0.122)
Logistic Regression	Gradient-DDP	0.933/0.850/0.979 (0.921 ± 0.054)
Logistic Regression	VLM (CLIP)	0.748/0.819/0.557 (0.708 ± 0.110)

**Table 6:** Classification performance (MS mild, MS moderate, MS severe) across different models, feature extraction methods, and MRI modalities.

Model	Feature Method	Mod.	Prec.	Rec.	F1	Acc.
SVM	Gradient-DDP	T1	0.80	0.80	0.80	0.80
		T2	0.97	0.97	0.97	0.96
		Flair	0.84	0.84	0.84	0.84
	VLM	T1	0.66	0.66	0.65	0.66
		T2	0.97	0.97	0.97	0.97
		Flair	0.63	0.62	0.62	0.62
KNN	Gradient-DDP	T1	0.83	0.79	0.80	0.79
		T2	0.85	0.84	0.83	0.84
		Flair	0.75	0.74	0.74	0.74
	VLM	T1	0.85	0.85	0.85	0.85
		T2	0.96	0.96	0.96	0.96
		Flair	0.56	0.56	0.56	0.57
LDA	Gradient-DDP	T1	0.83	0.81	0.82	0.81
		T2	0.89	0.89	0.89	0.89
		Flair	0.79	0.79	0.79	0.79
	VLM	T1	0.95	0.95	0.95	0.95
		T2	0.90	0.90	0.90	0.90
		Flair	0.61	0.51	0.51	0.51
Logistic Regression	Gradient-DDP	T1	0.81	0.67	0.66	0.67
		T2	0.97	0.97	0.97	0.96
		Flair	0.76	0.75	0.75	0.75
	VLM	T1	0.51	0.48	0.49	0.49
		T2	0.93	0.92	0.92	0.92
		Flair	0.76	0.75	0.75	0.75

**Table 7:** Confusion matrices for 3-class MS classification: (a) SVM and LDA models. (b) KNN and Logistic Regression models.

Model	Feature	Modality	Confusion Matrix
(a)			
SVM	Gradient-DDP	T1	$\left[\begin{array}{ccc} 144 & 7 & 0\\ 23 & 163 & 20\\ 0 & 45 & 78 \end{array}\right]$
		T2	$\left[\begin{array}{ccc} 142 & 4 & 1\\ 0 & 153 & 4\\ 0 & 8 & 168 \end{array}\right]$
		Flair	$\left[\begin{array}{ccc} 618 & 66 & 3\\ 100 & 684 & 74\\ 0 & 51 & 754 \end{array}\right]$
	VLM	T1	$\left[\begin{array}{ccc} 110 & 27 & 14\\ 34 & 146 & 26\\ 27 & 36 & 60 \end{array}\right]$
		T2	$\left[\begin{array}{ccc} 157 & 1 & 0\\ 1 & 148 & 6\\ 0 & 8 & 159 \end{array}\right]$
		Flair	$\left[\begin{array}{ccc} 506 & 189 & 92\\ 111 & 424 & 323\\ 40 & 179 & 586 \end{array}\right]$
LDA	Gradient-DDP	T1	$\left[\begin{array}{ccc} 133 & 27 & 4\\ 16 & 89 & 15\\ 0 & 29 & 167 \end{array}\right]$
		T2	$\left[\begin{array}{ccc} 143 & 1 & 0\\ 29 & 129 & 1\\ 0 & 20 & 157 \end{array}\right]$
		Flair	$\left[\begin{array}{ccc} 592 & 195 & 0\\ 119 & 642 & 97\\ 0 & 108 & 697 \end{array}\right]$
	VLM	T1	$\left[\begin{array}{ccc} 148 & 1 & 0\\ 8 & 153 & 3\\ 0 & 11 & 156 \end{array}\right]$
		T2	$\left[\begin{array}{ccc} 141 & 10 & 0\\ 17 & 184 & 5\\ 0 & 18 & 105 \end{array}\right]$
		Flair	$\left[\begin{array}{ccc} 330 & 442 & 15\\ 34 & 630 & 194\\ 15 & 507 & 283 \end{array}\right]$
(b)			
KNN	Gradient-DDP	T1	$\left[\begin{array}{ccc} 122 & 38 & 4\\ 15 & 101 & 4\\ 0 & 42 & 154 \end{array}\right]$
		T2	$\left[\begin{array}{ccc} 144 & 0 & 0\\ 4 & 116 & 6\\ 4 & 32 & 141 \end{array}\right]$
		Flair	$\left[\begin{array}{ccc} 657 & 130 & 0\\ 235 & 552 & 71\\ 12 & 190 & 603 \end{array}\right]$
	VLM	T1	$\left[\begin{array}{ccc} 135 & 16 & 0\\ 29 & 166 & 11\\ 0 & 18 & 105 \end{array}\right]$
		T2	$\left[\begin{array}{ccc} 145 & 4 & 0\\ 4 & 149 & 3\\ 0 & 9 & 166 \end{array}\right]$
		Flair	$\left[\begin{array}{ccc} 527 & 185 & 75\\ 187 & 420 & 251\\ 106 & 261 & 438 \end{array}\right]$
Logistic Regression	Gradient-DDP	T1	$\left[\begin{array}{ccc} 76 & 1 & 63\\ 0 & 73 & 92\\ 0 & 2 & 173 \end{array}\right]$
		T2	$\left[\begin{array}{ccc} 113 & 38 & 0\\ 7 & 195 & 4\\ 0 & 73 & 50 \end{array}\right]$
		Flair	$\left[\begin{array}{ccc} 564 & 222 & 1\\ 106 & 639 & 113\\ 0 & 180 & 625 \end{array}\right]$
	VLM	T1	$\left[\begin{array}{ccc} 52 & 85 & 14\\ 29 & 146 & 31\\ 2 & 83 & 38 \end{array}\right]$
		T2	$\left[\begin{array}{ccc} 141 & 2 & 1\\ 0 & 153 & 2\\ 0 & 8 & 169 \end{array}\right]$
		Flair	$\left[\begin{array}{ccc} 564 & 222 & 1\\ 106 & 639 & 113\\ 0 & 180 & 625 \end{array}\right]$

**Table 8:** Matthews Correlation Coefficient (MCC) for multi-class MS severity classification across MRI modalities.

Model	Feature Extraction	MCC (T1/T2/FLAIR) + Mean ± Std
SVM	Gradient-DDP	0.62/0.95/0.86 (0.81 ± 0.14)
SVM	VLM (CLIP)	0.31/0.94/0.43 (0.56 ± 0.27)
LDA	Gradient-DDP	0.66/0.83/0.78 (0.76 ± 0.07)
LDA	VLM (CLIP)	0.94/0.67/0.20 (0.60 ± 0.30)
KNN	Gradient-DDP	0.71/0.90/0.73 (0.78 ± 0.09)
KNN	VLM (CLIP)	0.83/0.94/0.32 (0.70 ± 0.27)
Logistic Regression	Gradient-DDP	0.52/0.55/0.68 (0.58 ± 0.07)
Logistic Regression	VLM (CLIP)	0.20/0.96/0.68 (0.61 ± 0.32)

**Table 9:** Comparative results of Multiple Sclerosis (MS) detection and progression studies using the 3D BrainWeb dataset.

Reference/Study	Method	Accuracy (%)	MRI Modality
Disease Detection (Literature)			
Yahia et al. (2018) [3]—Disease progression	3D-DDP + SVM	73.9	T1
Yahia et al. (2018) [3]—Noise robustness	3D-DDP + SVM	97.12 (T1)/94.21 (T2)	T1, T2
Yahia et al. (2018) [3]—Inhomogeneity robustness	3D-DDP + SVM	98.97 (T1)/93.24 (T2)	T1, T2
Yahia et al. (2023)—Disease progression [12]	DDP-NOP + SVM	93.9	T1
Salem et al. (2025) [33]—Disease identification	DDP-NOP + SVM	∼100 (T2)/83.33 (T1)	T1, T2
Disease Identification (This Study)			
This study	Gradient-DDP + SVM	90(T1)/98 (T2)/99 (Flair)	T1, T2
Disease Progression (This Study)			
This study	Gradient-DDP + SVM	80 (T1)/96 (T2)/84 (Flair)	T1, T2

## Data Availability

The data used in this study are publicly available. BrainWeb dataset is available at https://brainweb.bic.mni.mcgill.ca/brainweb (accessed on 15 January 2026). The MS–NonMS Classification (FLAIR) dataset is available on Kaggle at https://www.kaggle.com/datasets/farahmo/ms-nonms-classificationflair (accessed on 30 January 2026).
